# Visualizable vs. standard, non-visualizable steerable sheath for pulmonary vein isolation procedures: Randomized, single-centre trial

**DOI:** 10.3389/fcvm.2022.1033755

**Published:** 2022-11-16

**Authors:** Kristof Janosi, Dorottya Debreceni, Benedek Janosa, Botond Bocz, Tamas Simor, Peter Kupo

**Affiliations:** Heart Institute, Medical School, University of Pécs, Pécs, Hungary

**Keywords:** atrial fibrillation, pulmonary vein isolation (PVI), catheter ablation, visualizable steerable sheath, electroanatomical mapping system

## Abstract

**Introduction:**

Steerable sheaths (SSs) are frequently used to improve catheter contact during pulmonary vein isolation (PVI) procedures. A new type of visualizable (by electroanatomical mapping system) SS has become available in clinical treatment.

**Purpose:**

We aimed to compare procedural data of visualizable vs. non-visualizable steerable sheath assisted PVI procedures in patients with atrial fibrillation (AF).

**Methods:**

In this single-centre randomized study, we enrolled a total of 100 consecutive patients who underwent PVI due to AF.

**Results:**

A total of 100 patients were randomized into 2 groups (visualizable SS group: 50; non-visualizable SS group: 50). Acute ablation success was 100% and the rate of the first pass isolation were similar (92% vs. 89%; *p* = 0.88). Using visualizable SS, left atrial (LA) procedure time (53.1 [41.3; 73.1] min vs. 59.5 [47.6; 74.1] min.; *p* = 0.04), LA fluoroscopy time (0 [0; 0] s vs. 17.5 [5.5; 69.25] s; *p* < 0.01) and LA fluoroscopy dose (0 [0; 0.27] mGy vs. 0.74 [0.16; 2.34] mGy; *p* < 0.01) was significantly less, however, there was no difference in the total procedural time (90 ± 35.2 min vs. 99.5 ± 31.8 min; *p* = 0.13), total fluoroscopy time (184 ± 89 s vs. 193 ± 44 s; *p* = 0.79), and total fluoroscopy dose (9.12 ± 1.98 mGy vs. 9.97 ± 2.27 mGy; *p* = 0.76). Compared to standard, non-visualizable SS group, the number of radiofrequency ablations was fewer (69 [58; 80] vs. 79 [73; 86); *p* < 0.01) as well as total ablation time was reduced (1049 sec. [853; 1175] vs. 1265 sec. [1085; 1441]; *p* < 0.01) in the visualizable SS cohort. No major complications occurred in either group.

**Conclusion:**

Compared to the standard, non-visualizable SS, visualizable SS significantly reduces the left atrial procedure time, RF delivery and fluoroscopy exposure without compromising its safety or effectiveness in patients undergoing PVI procedures for AF.

## Graphical Abstract

Using visualizable or non-visualizable steerable sheaths for pulmonary vein isolation. 
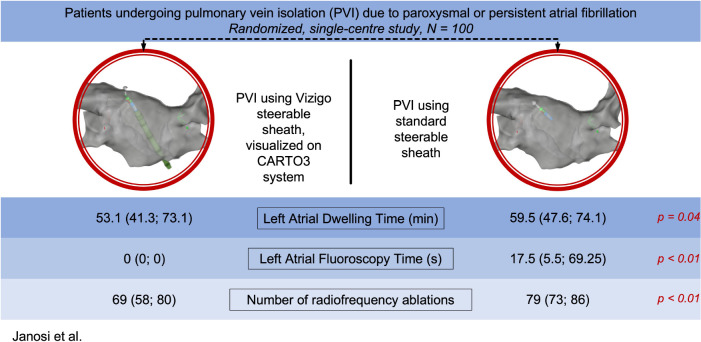

## Introduction

Atrial fibrillation (AF) is the most common arrhythmia, with a prevalence between 2 and 4% in adults (1). According to the most recent guidelines published by the European Society of Cardiology (ESC), in the management of AF, the primary indication for rhythm control strategy is to reduce AF-related symptoms and improve quality of life (2). Catheter ablation (CA) for AF is superior to antiarrhythmic drugs (AAD) for the maintenance of sinus rhythm (3–8).

The cornerstone of the AF ablation procedures is the complete electrical isolation of the pulmonary veins (2). To achieve pulmonary vein isolation (PVI) steerable sheaths (SS) are frequently used, which enables the operator to improve the contact and stability of the ablation catheter, which are crucial for effective lesion formation in the left atrial myocardium during point-by-point radiofrequency (RF) ablation (9–11).

**FIGURE 1:**
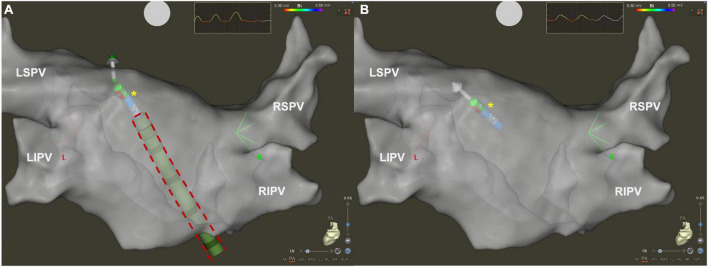
Three dimensional electroanatomical map of left atrium in posteroanterior view visualized by CARTO3 system. **(A)** Using visualizable steerable sheath (red dashed line), it is easier to understand spatial relationship between the ablation catheter (yellow asterisk) and the sheath. **(B)** Using standard steerable sheath, only the ablation catheter is visualized by the CARTO3 system. LIPV, left inferior pulmonary vein; LSPV, left superior pulmonary vein; RIPV, right inferior pulmonary vein; RSPV, right superior pulmonary vein.

Advance in technology can help to optimize procedural workflow and reduce radiation exposure for AF ablation procedures. A new type of SS (VIZIGO, Biosense Webster Inc., Irvine, CA) has become available in clinical treatment, which can be visualized by CARTO electroanatomical mapping system (Biosense Webster Inc., Irvine, CA, USA; Figure 1). VIZIGO can be visualized on the CARTO3 System based on advanced catheter location (ACL) technology. The sheath itself has an 8.5 French inner lumen, and it is bi-directional, allowing a 180 degrees deflection in both directions.

In our prospective randomized trial, we aimed to compare the procedural outcomes of patients undergoing PVI procedures performed by either visualizable or standard, non-visualizable SSs.

## Methods

### Study patients

In our prospective singe-centre trial, 100 consecutive patients undergoing PVI procedure for paroxysmal or persistent AF were randomized into visualizable (VIZIGO, Biosense Webster Inc., Irvine, CA) or standard, non-visualizable (Agilis™ NxT, St. Jude Medical, St. Paul, MN, USA) SS groups.

Exclusion criteria were (a) previously performed PVI procedure; (b) additional ablations beyond PVI (including any left or right atrial ablations); and (c) age under 18 years.

All procedures were accomplished by the same expert electrophysiologist. The protocol of the trial is in accordance with the Declaration of Helsinki and the study protocol was approved by the regional ethics committee. All patients provided written informed consent for the study protocol.

### Study protocol

During the procedures, fentanyl ± midazolam was used to achieve a conscious sedation. After local anesthesia, following vascular ultrasound guided femoral venous puncture one decapolar steerable catheter (Dynamic Deca, Bard Electrophysiology, Lowell, MA, USA) was positioned in the coronary sinus (CS). After intracardiac echocardiography (ICE)-guided double transseptal puncture a multipolar, steerable, circular mapping catheter (Lasso NAV, Biosense Webster Inc., Diamond Bar, CA, USA) was inserted in the left atrium *via* SL0. Besides, a contact force (CF)–sensing ablation radiofrequency (RF) ablation catheter (Navistar Thermocool SmartTouch ST NAV, Biosense Webster Inc., Diamond Bar, CA, USA) was positioned into the left atrium through either visualizable or standard, non-visualizable SSs. A fast anatomical map of the left atrium was performed with the Lasso NAV catheter supported by CARTO electroanatomical mapping system (Biosense Webster Inc., Diamond Bar, CA, USA). Ablation catheter was set in a power-controlled mode with a maximum power of 45 W for anterior and 35 W for posterior wall using a maximum temperature of 43°C.

During RF ablations, CARTO VISITAG™ Module was used with minimum stability time of 4 s and maximum location stability range of 2.5 mm. Visitag Surpoint (i.e., ablation index) was applied with targets of 350 at the posterior wall and 450 at the anterior wall. Target interlesion distance was <5 mm. Point-by-point ablation technique was used, contact force (CF) and impedance was monitored in real time. CF was held between 5 and 15 g during ablation.

During the ablations, to blind the operator to the presence or absence of first-pass isolation, Lasso catheter was positioned in the contralateral pulmonary veins. Intravenous unfractionated heparin was administered immediately after the first transseptal puncture, and an activated clotting time of >300 s was held for the whole duration of the procedure. The procedural endpoint of the ablation was obtained if all PVs were isolated. Based on our institutional protocol, only PV isolation was performed even in persistent AF cases.

Procedure time was defined as the time from the first femoral vein puncture until the removal of the catheters. Left atrial time was measured from the end of the TS until the withdrawal of the sheaths from the left atrium. Fluoroscopy time and radiation dose were automatically measured by the fluoroscopy system. Total number of the RF applications and total ablation time were recorded by the EP recording system (CardioLab, GE Healthcare).

The occurrence of major complications (i.e., vascular complications, pericardial effusion, cardiac tamponade, stroke, or atrio-esophageal fistula) were systematically evaluated during the whole hospitalization.

### Statistical analysis

Data were analyzed according to their normal distribution on the Kolmogorov–Smirnov goodness-of-fit test. Continuous data were presented as the mean ± SD or median (interquartile range, IQR), as appropriate while categorical variables are presented as absolute numbers and percentages. For comparisons, chi-square test, *t*-test, and Mann–Whitney U test were applied as appropriate. A *p*-value < 0.05 was considered statistically significant in all analyses. Statistical analyses were performed using SPSS 24 software (SPSS, Inc., Chicago, IL, USA).

## Results

A total of 100 patients were randomized into visualizable or non-visualizable SS groups. No intra- or postprocedural patient exclusion was applied. We did not find any significant difference in the baseline characteristics of the study population between the groups (male sex: 80% vs. 70%, *p* = 0.25; age: 56.0 ± 17.4 vs. 58.2 ± 13.1 years, *p* = 0.74, Table 1).

**TABLE 1:** Clinical characteristics of the study population.

	Visualizable steerable sheath group (*n* = 50)	Non-visualizable steerable sheath group (*n* = 50)
Age, years	56.0 ± 17.4	58.2 ± 13.1
Male (%)	40 (80.0)	35 (70.0)
Paroxysmal AF (%)	37 (74.0)	39 (78.0)
Persistent AF (%)	13 (26.0)	11 (22.0)
Hypertension (%)	39 (78.0)	35 (70.0)
Diabetes mellitus (%)	7 (14.0)	10 (20.0)
Prior stroke/TIA (%)	1 (2.0)	2 (4.0)
Heart failure (%)	2 (4.0)	1 (2.0)
Chronic kidney disease (%)	3 (6.0)	4 (8.0)
Left atrial diameter, mm	52.0 ± 10.6	55.0 ± 12.2

AF, atrial fibrillation; TIA, transient ischemic attack.

In all 100 cases PVs were isolated, thus procedural endpoint was achieved, and acute procedural success was 100%. The rate of the first pass isolation were similar (92% vs. 89%; *p* = 0.88). Total procedural time did not differ between visualizable vs. non-visualizable SS groups (90 ± 35.2 min. vs. 99.5 ± 31.8 min.; *p* = 0.97). Using visualizable SS, left atrial procedure time (53.1 [41.3; 73.1] min vs. 59.5 [47.6; 74.1] min.; *p* = 0.04), left atrial fluoroscopy time (0 [0; 0] s vs. 17.5 [5.5; 69.25] s; *p* < 0.01) and left atrial fluoroscopy dose (0 [0; 0.27] mGy vs. 0.74 [0.16; 2.34] mGy; *p* < 0.01) was significantly less, however, there was no difference in total fluoroscopy time (184 ± 89 s vs. 193 ± 44 s; *p* = 0.79), and total fluoroscopy dose (9.12 ± 1.98 mGy vs. 9.97 ± 2.27 mGy; *p* = 0.76). More procedures were performed fluoroless following the transseptal puncture in the visualizable SS group (88.0% vs. 16.0%, *p* < 0.001).

Compared to non-visualizable SS, the number of radiofrequency ablations was fewer (69 [58; 80] vs. 79 [73; 86]; *p* < 0.01) as well as total ablation time was reduced (1049 s. [853; 1175] vs. 1265 s. [1085; 1441]; *p* < 0.01) in the visualizable SS cohort. No major complications occurred in either group. We summarized our results in Table 2.

**TABLE 2:** Procedural parameters in the study population.

	Visualizable steerable sheath group (*n* = 50)	Non-visualizable steerable sheath group (*n* = 50)	*P-value*
Total procedure time (min)	90 ± 35.2	99.5 ± 31.8	0.97
Left atrial procedure time (min)	53.1 (41.3; 73.1)	59.5 (47.6; 74.1)	0.04
Total fluoroscopy time (s)	184 ± 89	193 ± 44	0.79
Total fluoroscopy dose (mGy)	9.12 ± 1.98	9.97 ± 2.27	0.76
Left atrial fluoroscopy time (s)	0 (0; 0)	17.5 (5.5; 69.25)	<0.01
Left atrial fluoroscopy dose (mGy)	0 (0; 0.27)	0.74 (0.16; 2.34)	<0.01
Fluoroless procedure after transseptal puncture	44 (88.0)	8 (16.0)	<0.001
Acute ablation success (%)	50 (100)	50 (100)	1
Number of radiofrequency ablations	69 (58; 80)	79 (73; 86)	<0.01
Total ablation time (s)	1049 (853; 1175)	1265 (1085; 1441)	<0.01
First pass isolation (%)	92%	89%	0.88
Major complications (n)	0	0	N.A.

n.s., non-significant; N.A., not available.

We performed statistical analysis separately for persistent AF cases. Results showed similar data as the overall cohort, however, there was no difference between the groups in the left atrial procedure time (54.8 [44.3; 59.0] min vs. 66.9 [50.0; 73.7] min, *p* = 0.23) and the total fluoroscopy time was reduced in the visualizable SS group (182 ± 52 s vs. 244 ± 84 s, *p* = 0.02). Data shown in Table 3.

**TABLE 3:** Procedural parameters in persistent atrial fibrillation cases.

	Visualizable steerable sheath group (*n* = 13)	Non-visualizable steerable sheath group (*n* = 11)	*P-value*
Total procedure time (min)	100 ± 19.0	103 ± 21.5	0.36
Left atrial procedure time (min)	54.8 (44.3; 59.0)	66.9 (50.0; 73.7)	0.23
Total fluoroscopy time (s)	182 ± 52	244 ± 84	0.02
Total fluoroscopy dose (mGy)	14.4 ± 11.2	17.6 ± 12.4	0.43
Left atrial fluoroscopy time (s)	0 (0; 0)	25 (6; 77)	<0.001
Left atrial fluoroscopy dose (mGy)	0 (0; 0)	1.13 (0.16; 1.74)	0.02
Fluoroless procedure after transseptal puncture	11 (84.6)	2 (18.2)	<0.01
Acute ablation success (%)	50 (100)	50 (100)	1
Number of radiofrequency ablations	68 (55; 78)	79 (73; 86)	0.04
Total ablation time (s)	951 (829; 1095)	1265 (1085; 1441)	0.04
First pass isolation (%)	92%	82%	0.44
Major complications (n)	0	0	N.A.

N.A., not available.

## Discussion

Catheter ablation for AF is the most frequently performed ablation procedure worldwide. The integration of novel technologies in procedural workflows can help to achieve significant reductions in fluoroscopy exposure and procedural times for PVI. During these procedures, transseptal sheaths are routinely used to reduce procedural time and improve acute and long-term success rate. SSs can improve the contact and stability of the ablation catheter, thus were found superior compared to fixed sheaths (12).

The novel type SS, unlike the standard SS, can be visualized in CARTO3 navigation system with the help of electrodes and the magnetic sensors of the ablation catheter. The visualization of the sheath helps to determine the spatial relationship between the ablation catheter and the sheath during catheter manipulation. However, to date, limited scientific data (only from observation studies) are available evaluating the effect of using visualizable SS for AF ablation procedures.

In an observational study performed by Guo et al. visualizable SS was compared to fixed sheath in patients who underwent PVI procedures for paroxysmal AF, found that the novel type SS for CA reduced radiation exposure, moreover, it significantly improved CF and initial PVI rate. Total procedural time was shorter with the use of visualizable SS, however, left atrial procedural time did not differ between the groups (13).

A recently published observational study by Rajendra et al. compared PVI procedures performed by visualizable SS sheaths vs. a cohort, where no transseptal sheaths were used. They found no difference in clinical effectiveness, however, visualizable SS helped to improve catheter stability and to reduce ablation time, besides, more procedures could be performed without applying fluoroscopy (14).

In our single-centre randomized trial we found that use of visualizable SS reduced left atrial procedural time, left atrial fluoroscopy time, total ablation time and number of RF applications, while effectiveness and safety was equal compared to the standard, non-visualizable SS. These results could be due to the improved catheter stability, however, we did not collect data about contact force values. Importantly, using visualizable SS, in 44 out of 50 cases, we performed the procedure fluoroless following the transseptal puncture, which also proved to be more common compared to the standard, non-visualizable SS group. Moreover, the use of visualizable SS reduced total fluoroscopy time in persistent AF cases compared to non-visualizable SS group.

During an AF ablation procedure, the average patient fluoroscopy dose approximates 15 mSv, which increases the absolute lifetime risk of fatal cancer for an adult by 0.075% (15). Besides, annual radiation exposure of interventional cardiologists and electrophysiologists may even reach an effective dose of 5 mSv yearly (16). Although this risk can be reduced by applying various forms of radiation protection and the “as low as reasonably achievable” (ALARA) principle, it remains still of great importance. Furthermore, the wearing of lead aprons is associated with a higher rate of work-related musculoskeletal pain (17–19).

The use of EAM systems besides ICE can efficiently help in reducing the radiation exposure without compromising the safety and efficacy of the ablation procedures. With the implementation of visualizable VIZIGO sheath, the fluoroscopy exposure can be reduced effectively, thus it can support to achieve zero- or minimal fluoroscopic AF ablations.

Based on the results of the meta-analysis performed by Huang et al., comparing conventional fluoroscopy vs. low/zero-fluoroscopy PVI procedures, similar clinical efficacy and safety can be reached by the adoption of alternative imaging modalities such as 3D EAM systems, force-sensing ablation catheters and ICE. Moreover, low/zero-fluoroscopy approach was associated with shorter procedure time besides reduced fluoroscopy time and exposure (20). Visualizable SS was not used in either involved studies, however, considering the available scientific data, application of these types of SSs could help in the feasibility of the low/zero-fluoroscopy approach and improve procedural outcomes of PVI procedures.

## Limitations

Our results should be interpreted with the careful consideration of the following limitations. Firstly, this was a randomized, single-centre, single-operator study with a limited number of patients enrolled, which may limit its generalizability. Secondly, data about contact force parameters were not available. Finally, our study does not provide data on whether the long-term results are influenced by the type of SS. Multicentre trials are required to assess and to improve clinical outcomes with visualizable SSs.

## Conclusion

Compared to the standard, non-visualizable SS, visualizable SS significantly reduces the left atrial procedure time, RF delivery and fluoroscopy exposure without compromising its safety or effectiveness in patients undergoing PVI procedures for AF.

## Data availability statement

The datasets presented in this article are not readily available because of Hungarian legal regulations. Requests to access the datasets should be directed to PK, peter.kupo@gmail.com.

## Ethics statement

The studies involving human participants were reviewed and approved by the Regional Ethics Committee of University of Pécs. The patients/participants provided their written informed consent to participate in this study.

## Author contributions

KJ, PK, and TS contributed to the concept and design of the study. PK performed the statistical analysis. KJ, PK, and DD wrote the different sections of the manuscript. DD, BB, and BJ contributed to the data collection and making of the figure and tables. All authors contributed to the manuscript revision, read, and approved the submitted version.
